# SydR, a redox-sensing MarR-type regulator of *Sinorhizobium meliloti*, is crucial for symbiotic infection of *Medicago truncatula* roots

**DOI:** 10.1128/mbio.02275-24

**Published:** 2024-10-31

**Authors:** Fanny Nazaret, Davoud Farajzadeh, Joffrey Mejias, Marie Pacoud, Anthony Cosi, Pierre Frendo, Geneviève Alloing, Karine Mandon

**Affiliations:** 1 Université Côte d'Azur, INRAE, CNRS, ISA, Sophia-Antipolis, France; 2 Azarbaijan Shahid Madani University, Tabriz, Iran; 3 IRD, CIRAD, Université Montpellier, Plant Health Institute, Montpellier, France; Duke University School of Medicine, Durham, North Carolina, USA

**Keywords:** redox signaling, MarR family regulator, bacterial infection, *Sinorhizobium meliloti*, nitrogen-fixing symbiosis

## Abstract

Rhizobia associate with legumes and induce the formation of nitrogen-fixing nodules. The regulation of bacterial redox state plays a major role in symbiosis, and reactive oxygen species produced by the plant are known to activate signaling pathways. However, only a few redox-sensing transcriptional regulators (TRs) have been characterized in the microsymbiont. Here, we describe SydR, a novel redox-sensing TR of *Sinorhizobium meliloti* that is essential for the establishment of symbiosis with *Medicago truncatula*. SydR, a MarR-type TR, represses the expression of the adjacent gene SMa2023 in growing cultures, and this repression is alleviated by NaOCl, *tert-*butyl hydroperoxide, or H_2_O_2_ treatment. Transcriptional *psydR-gfp* and *p*SMa2023-*gfp* fusions, as well as gel shift assays, showed that SydR binds two independent sites of the *sydR*-SMa2023 intergenic region. This binding is redox-dependent, and site-directed mutagenesis demonstrated that the conserved C16 is essential for SydR redox sensing. The inactivation of *sydR* did not alter the sensitivity of *S. meliloti* to NaOCl, *tert*-butyl hydroperoxide, or H_2_O_2_, nor did it affect the response to oxidants of the roGFP2-Orp1 redox biosensor expressed within bacteria. However, *in planta*, Δ*sydR* mutation impaired the formation of root nodules. Microscopic observations and analyses of plant marker gene expression showed that the Δ*sydR* mutant is defective at an early stage of the bacterial infection process. Altogether, these results demonstrated that SydR is a redox-sensing MarR-type TR that plays a key role in the regulation of nitrogen-fixing symbiosis with *M. truncatula*.

**IMPORTANCE:**

The nitrogen-fixing symbiosis between rhizobia and legumes has an important ecological role in the nitrogen cycle, contributes to nitrogen enrichment of soils, and can improve plant growth in agriculture. This interaction is initiated in the rhizosphere by a molecular dialog between the two partners, resulting in plant root infection and the formation of root nodules, where bacteria reduce the atmospheric nitrogen into ammonium. This symbiosis involves modifications of the bacterial redox state in response to reactive oxygen species produced by the plant partner. Here, we show that SydR, a transcriptional regulator of the *Medicago* symbiont *Sinorhizobium meliloti*, acts as a redox-responsive repressor that is crucial for the development of root nodules and contributes to the regulation of bacterial infection in *S. meliloti/Medicago truncatula* symbiotic interaction.

## INTRODUCTION

Bacteria have to cope with environmental variations either as free-living microorganisms or during interaction with eukaryotic organisms. The success of this adaptation depends on their ability to coordinate changes in gene expression and maintain cellular homeostasis. In particular, bacteria have evolved different transcriptional regulators able to sense variations in the levels of redox-active compounds, such as reactive oxygen species (ROS) and reactive nitrogen species (RNS), known as key signaling molecules. Thereby, redox-responsive regulators modulate the expression of target genes involved in bacterial response to changes in redox state and in ROS-RNS-regulated cell processes (1). The important role of ROS has been particularly highlighted in beneficial and pathogenic interactions between plants and microorganisms (2–4). Accordingly, the production of ROS in plants and antioxidant defense in bacteria are major determinants of the outcome of the interactions.

Soil rhizobacteria are able to reduce atmospheric nitrogen (N_2_) into ammonia in symbiosis with a wide range of legumes, enabling them to sustain plant growth in nitrogen-limited environments. Thanks to this biological nitrogen fixation (BNF), the symbiotic bacteria contribute to nearly half of the nitrogen input in crop soils, giving legume plants an agronomic advantage (5). BNF occurs in a newly emerged plant organ called a nodule. Both rhizobia and host plants exhibit narrow specificity, and the interaction is initiated by an exchange of molecular signals. Flavonoids released into the rhizosphere by the host plant induce bacterial secretion of lipochito-oligosaccharides, the nodulation factors (NFs). NFs, in turn, trigger signal transduction pathways in plants, leading to activation of central regulators and subsequent reprogramming of root cortical cells for nodule organogenesis and bacterial infection (6). In *Medicago* sp., the root hairs curl to form an infection pocket where entrapped bacteria divide. Then, the bacteria progress toward plant cortical cells, within a host tubular structure emerging from the infection pocket called infection thread (IT). Finally, the bacteria are released inside plant cells and differentiate into nitrogen-fixing bacteroids.

ROS play an important role during all steps of symbiosis development, and changes in ROS concentration have been detected from the the early stages of interaction to mature nodules (7). A recent analysis using redox biosensors has established that the microsymbiont maintains a reduced state inside IT and undergoes an oxidative upshift during bacteroid differentiation (8). Moreover, several genes involved in the antioxidant defense of the microsymbiont are known to be required for optimal symbiosis (9). The search for central redox-sensing regulators involved in the control of the legume-rhizobia symbiosis has been undertaken for several years. In bacteria, most of the redox-sensing regulators belonging to the LysR and MarR (Multiple antibiotic resistance Regulator) families are involved in various processes (10). Their activity is often regulated via oxidative post-translational modification of specific cysteines. This mechanism enables redox-sensing regulators to rapidly trigger activation or inactivation of their target promoters. In *Sinorhizobium meliloti*, the conserved LysR-like regulator OxyR contributes significantly to the response to H_2_O_2_ in growing cultures (11, 12). However, no symbiotic phenotype has been associated with an *oxyR*-inactivated mutant. Following a large-scale mutagenesis approach, another LysR-like redox-sensing regulator, LsrB, was identified for its important role in the efficiency of *S. meliloti*/*Medicago sativa* interaction (13). A *lsrB* deletion mutant induces ineffective, early senescing nodules with abnormal bacteroid differentiation (14). Besides LysR-like regulators, members of the MarR family are widely distributed in bacteria and archaea (15). The redox-responsive MarR-type regulators are generally repressors that are inactivated upon oxidation, triggering target gene expression. Among them, the MarR/OhrR proteins respond more particularly to peroxides (10). In *S. meliloti*, the expression of *ohr* in symbiosis is regulated by OhrR, but neither *ohrR* nor *ohr* inactivation affects symbiosis efficiency (16). Finally, while this work was in progress, Zhang et al. (17) published that a deletion in SMa2020, an OhrR-like encoding gene, reduced IT formation and plant growth with no reported effect on the number of nodules in *S. meliloti*/*M. sativa* interaction.

In this work, the redox response of MarR-type candidates in *S. meliloti* was analyzed. Two of them, encoded by SMc00146 and SMa2020 genes, were found to be involved in the control of sodium hypochlorite (NaOCl)-inducible gene expression. SMa2020 encodes a transcriptional repressor containing a redox-sensitive cysteine. In contrast with the results observed during the *S. meliloti*/*M. sativa* interaction, the inactivation of SMa2020 (called SydR for symbiosis redox regulator) resulted in the early abortion of bacterial infection with a drastic decrease in nodule number during the interaction with *Medicago truncatula*.

## RESULTS

### Identification of redox-sensing MarR-type regulators in *S. meliloti*

**Fig 1:**
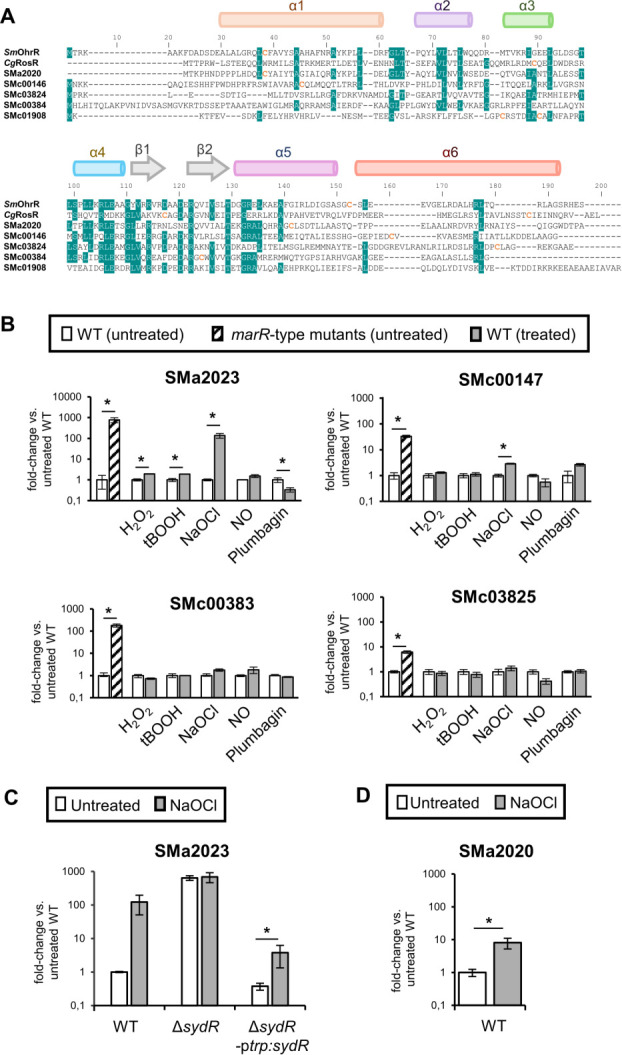
SydR is a new MarR-type redox-sensing repressor. (**A**) Sequence alignment of MarR-type regulators. The alignment between *S. meliloti* OhrR and SMa2020, SMc00146, SMc03824, SMc00384, SMc01908 products, and *Corynebacterium glutamicum* RosR, was generated using Clustal-W. Secondary structure elements were predicted from the sequence of SMa2020 product using Jalview (23). The HTH domain corresponds to helices α3 and α4, the wing to β-strands β1 and β2, and the dimerization region is formed by helices α1, α5, and α6. Cysteine residues are indicated in orange. Blue shading indicates an identity ≥70% at that position. (**B**) Analysis of MarR-type-mediated gene expression. RT-qPCR analysis of the expression of SMa2023, SMc00147, SMc00383, and SMc03825, considered as potential target genes of SMa2020, SMc00146, SMc00384, and SMc03824 products, respectively. The expression of each gene was analyzed in wild-type strain (WT) and related *marR*-type mutant under control condition (untreated) and in WT challenged with H2O2, tBOOH, NaOCl, NO or plumbagin (treated). (**C**) The induction of SMa2023 expression by NaOCl is SydR-dependent. RT-qPCR analysis of SMa2023 expression in WT, Δ*sydR* (SMa2020-inactivated mutant) and Δ*sydR-*p*trp:sydR* strains under control conditions (untreated) or challenged with NaOCl. (**D**) SMa2020 expression is induced by NaOCl. RT-qPCR analysis of SMa2020 expression in the WT strain under control condition (untreated) or challenged with NaOCl. (**B–D**) For each condition, transcription levels were normalized to those in untreated WT. The values shown are the means ± SEM of three independent experiments. Student’s *t* test was used to assess the statistical significance (*P < 0.05).

The annotation of *S. meliloti* genome (*S. meliloti* 2011 genome website, https://iant.toulouse.inra.fr) allowed the identification of 10 genes encoding MarR-type proteins with one or more cysteine(s) (Table S1). Three *S*. *meliloti marR-*type genes have already been studied, i.e., SMc00098 encoding OhrR, SMc01945 encoding Cpo, and SMb21317 encoding WggR (16, 18, 19). Of the seven candidates not yet characterized, SMc00562 and SMc04052 were ruled out because their expression is barely detected in nodules according to Roux et al. (20). Based on Clustal-O multiple alignment analyses with predicted secondary structure (Jalview) (21), the sequences of proteins encoded by the remaining five candidates display a typical wHTH (wing helix-turn-helix) DNA-binding domain in the central part of the protein and a largely helical dimerization region (Fig. 1A) (22). These features are typical of MarR-type regulators, which bind as a homodimer to palindromic sites (15). On the other hand, cysteines of SMc01908 product are located on DNA-binding α helix, which is unlike the localization of redox-sensitive cysteines in most MarR-type redox-regulators (22). Thus, subsequent analyses were conducted with SMa2020, SMc00146, SMc00384, and SMc03824.

Most redox-sensing MarRs are transcriptional repressors inactivated by redox-active compounds such as ROS, RNS, or NaOCl. To investigate the role of the four candidates in regulating gene expression, we identified a putative target gene in close vicinity of each MarR-type encoding gene (see Fig. S1A) and first examined their basal expression depending on *marR*-type gene integrity. To this end, mutants in SMa2020, SMc00146, SMc00384, and SMc03824 were constructed. Comparative RT-qPCR experiments were performed using total RNA extracted from WT and mutant bacteria grown in an M9 medium. As shown in Fig. 1B, a strong increase in specific mRNA level was obtained in the mutants as compared to the WT strain (with a fold change of 6, 33, 180, and 773 for SMc03825, SMc00147, SMc00383, and SMa2023, respectively). These results suggest that each selected *marR-*type gene encodes a repressor that inhibits the expression of proposed target gene under basal conditions.

To test whether the repression by MarR-type proteins was dependent on redox sensing, bacterial cultures were exposed to various ROS and redox-active compounds, and the effect of treatments on target gene expression was investigated. Sublethal concentrations of H_2_O_2_, *tert*-butyl hydroperoxide (tBOOH), spermine NONOate (a nitric oxide generator), NaOCl, and plumbagin (an anion superoxide generator) capable of inducing gene expression were first determined, by using oxidative stress-inducible marker genes (Fig. S1B). Then, the same concentrations of oxidants were applied to analyze the expression of MarR-type target genes (Fig. 1B). SMc00383 and SMc03825 expression remained unchanged regardless of the added oxidant. In contrast, the expression of SMc00147 was increased threefold by NaOCl, while the SMa2023 was induced 137-fold by NaOCl, twofold by H_2_O_2_ or tBOOH, and reduced threefold by plumbagin addition. Therefore, the repression of SMc00147 and SMa2023 transcription can be alleviated by the addition of oxidants, supporting the hypothesis that SMc00146 and SMa2020 are redox-responsive regulators. Considering the higher induction of SMa2023 compared to SMc00147, we focused on the characterization of SMa2020, which was named SydR for symbiosis redox regulator.

To further analyze the relationship between SydR (SMa2020) and the NaOCl-induced expression of SMa2023, the SMa2023 expression level was determined in WT, Δ*sydR* (SMa2020 defective mutant) and in a Δ*sydR* strain expressing *sydR* under the control of the constitutive promoter p*trp* (Δ*sydR*-p*trp:sydR*) (Fig. 1C). A similar expression level was observed in both untreated and NaOCl-treated Δ*sydR* mutant, showing that the redox regulation of SMa2023 was lost in this strain. In contrast, the redox regulation of SMa2023 was recovered in the complemented strain Δ*sydR*-p*trp:sydR*. The *sydR* expression was also shown to be regulated by NaOCl, although to a lesser extent (13-fold versus 123-fold for SMa2023; Fig. 1C and D). Altogether, these data establish that the NaOCl-induced expression of SMa2023, and most probably *sydR*, is controlled by SydR.

### Specific binding of SydR to the *sydR*-SMa2023 intergenic region

**Fig 2:**
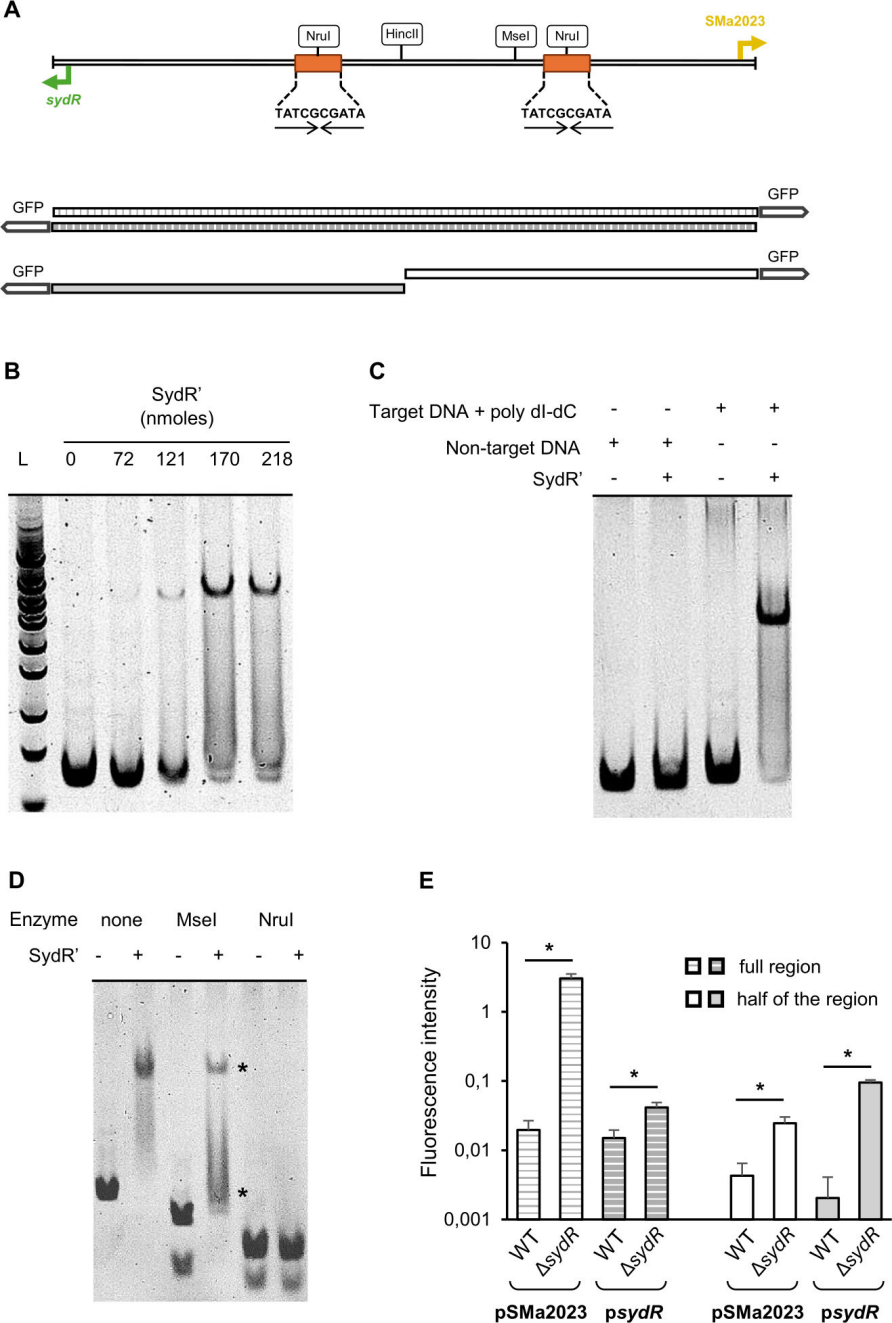
SydR binds with high specificity to the promoter region of *sydR* and SMa2023. (**A**) Restriction map of the 144 bp *sydR*-SMa2023 intergenic region. The transcription start sites (+1, bent arrows) and inverted repeat motifs (arrows) are indicated. The large arrow at each end of the sequence represents the translation initiation codon (ATG) of *sydR* or SMa2023. A schematic representation of transcriptional fusions with *gfp* and the entire s*ydR*-SMa2023 intergenic region or half of the region is designed. (B–D) EMSA analysis of SydR DNA-binding activity. (**B**) The *sydR*-SMa2023 intergenic region was incubated with increasing concentrations of SydR′, and the mixtures were separated in a polyacrylamide non-denaturing gel. (**C**) Non-specific DNA (an internal fragment of SMa2019), or specific DNA (*sydR*-SMa2023 intergenic region) in the presence of poly(dI-dC) added as competitor in a 1:1 ratio, was incubated in the presence (+) or not (−) of SydR′. (**D**) The *sydR*-SMa2023 intergenic region and its restriction fragments produced by MseI or NruI digestion were incubated with SydR′. Upshift bands are indicated by stars. (**E**) Transcriptional fusions with *gfp* and either the entire *sydR*-SMa2023 intergenic region or half of the region were expressed in *S. meliloti*. The measurements of green fluorescent protein (GFP) fluorescence in WT and Δ*sydR* backgrounds were expressed per bacterial quantity (OD_600_ unit). The values shown are the means ± SEM of three independent experiments. Student’s *t* test was used to assess the statistical significance (*P < 0.05).

To test the direct regulation of SMa2023 and *sydR* by SydR, we analyzed the interaction between the *sydR*-SMa2023 intergenic region, and a His-tagged recombinant SydR (SydR′) purified in the presence of dithiothreitol (DTT) (Fig. 2). The direct binding of SydR′ to *sydR*-SMa2023 intergenic region was detected by electrophoretic mobility shift assay (EMSA) with SydR′ and a 144-bp specific DNA fragment encompassing the entire region between *sydR* and SMa2023 ORFs (Fig. 2A). The addition of increasing concentrations of SydR′ resulted in a band up-shift in a dose-dependent manner (Fig. 2B). The interaction of SydR′ with target DNA was conserved in the presence of poly(dI-dC) DNA used as a competitor. In addition, SydR′ DNA binding was not observed when non-target DNA (an internal fragment of SMa2019) was used, demonstrating the specificity of the DNA-binding activity of SydR′ (Fig. 2C). Two identical perfect inverted repeat (IR) sequences (TATCGCGATA) were discovered in the *sydR*-SMa2023 intergenic region (Fig. 2A). Each is located upstream of the transcription start site of *sydR* and SMa2023 (the +1 position was previously defined by Schlüter et al. [24]), suggesting potential binding sites for the SydR dimer. To prove this, the SydR DNA binding was examined by EMSA with SydR′ and digested fragments of the *sydR*-SMa2023 intergenic region (Fig. 2D). A cleavage site for MseI is located between the two IRs, while NruI cleaves each of them (Fig. 2A). Digestion of target DNA by MseI led to two upshifted bands, whereas disruption of the IRs by NruI prevented any upshift (Fig. 2D). These data strongly suggest that SydR is capable of directly regulating SMa2023 and *sydR* by binding to the two motifs identified in the *sydR*-SMa2023 intergenic region.

The effectiveness of SydR binding to each site was further analyzed by using *gfp* transcriptional fusions under the control of the complete SMa2023-*sydR* intergenic region or under one-half of the intergenic region, covering either *sydR* or Sma2023 promoter region (Fig. 2A). The fusions were integrated into the genome of WT and Δ*sydR* strains, and the GFP-associated fluorescence was determined by fluorimetry (see Materials and Methods). For the four fusions, a higher fluorescence intensity was observed in Δ*sydR* versus WT background (Fig. 2E). These data showed that each half of the SMa2023-*sydR* intergenic region enables SydR to repress gene expression and confirmed the binding of SydR to two separate motifs present in *sydR* and in SMa2023 promoter regions.

### Inhibition of SydR activity occurs via oxidation of the redox-sensing C16

**Fig 3:**
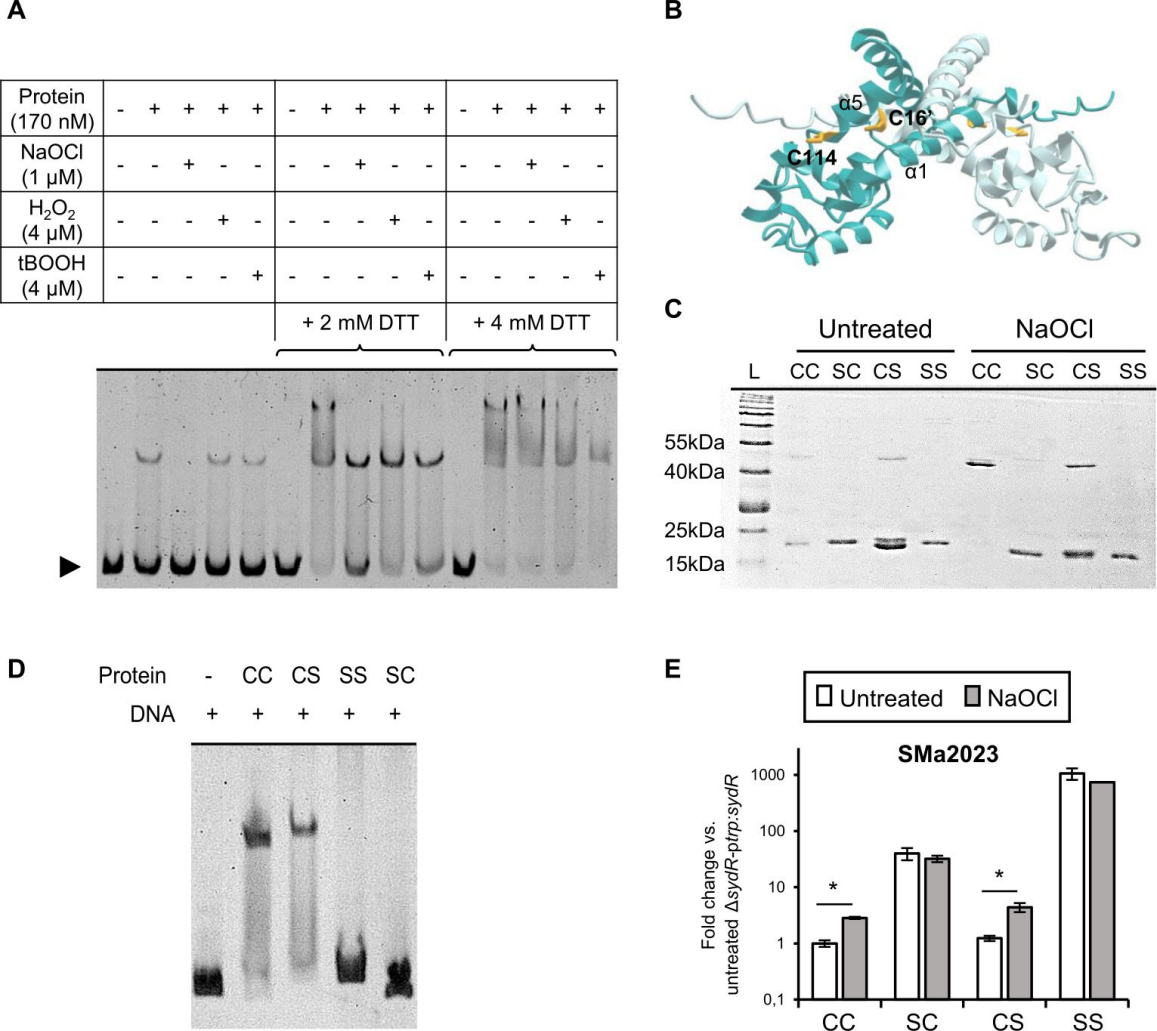
SydR-binding activity is inhibited by oxidation and depends on the redox-active cysteine C16. (**A**) EMSA with DNA (*sydR*-SMa2023 intergenic region) SydR′ in 0.5 mM DTT buffer treated or not with various oxidants was performed at 25°C for 25 min, followed by an additional 25 min incubation with 2 or 4 mM DTT as indicated. (**B**) Three-dimensional structural model of SydR in homodimeric configuration, generated using AlphaFold Protein Structure Database (https://colab.research.google.com/github/sokrypton/ColabFold/blob/main/AlphaFold2.ipynb). One subunit is colored turquoise-blue, and the other subunit is colored pale blue. Cysteines are shown as yellow sticks. C16 is predicted to be located at the dimer interface, and C114 to be exposed at the surface, like C22 and C127 in *Xanthomonas campestris* OhrR (25). Non-reducing SDS-page (**C**) and EMSA (**D**) using SydR′ WT protein (CC) and mutant variants harboring the C16S (SC), C114S (CS), and C16S&C114S (SS) mutations, treated with DTT then, when indicated, with NaOCl. (**E**) RT-qPCR analysis of SMa2023 expression in Δ*sydR*-p*trp:sydR* strain and Δ*sydR* derivatives encoding SydR mutant variants (SC, CS, or SS). The values shown are the means ± SEM of three independent experiments. Student’s *t* test was used to assess the statistical significance (*P < 0.05).

The sensitivity of SydR DNA-binding activity to oxidation was analyzed *in vitro* (Fig. 3A). SydR′ was subjected to oxidation by H_2_O_2_, tBOOH, or NaOCl application. As shown in Fig. 3A, SydR′ was bound to DNA in control condition as well as after treatment with H_2_O_2_ or tBOOH (4 μM). In contrast, SydR' underwent oxidative inhibition with 1 μM NaOCl. Subsequent incubation of the different samples with 2 or 4 mM DTT, increased SydR' binding, resulting in partial or total disappearance of free DNA fragment in Fig. 3A, lanes 7–10 and 12–15, respectively. These data specifically showed the complete reversibility of SydR' activity impairment following NaOCl oxidation. Thus the ability of SydR to bind DNA depends on the redox state of the protein, most likely via redox-sensitive cysteine(s). Besides, two DNA complexes were observed after prolonged incubation with reduced SydR′, most likely due to non-specific binding at high SydR/DNA ratio.

SydR contains two cysteines per monomer, C16 and C114, located on the N-terminal helix α1 and on the helix α5, respectively (Fig. 1A). The N-terminus cysteine is conserved in *S. meliloti* OhrR and other members of the MarR/OhrR family such as *X. campestris* OhrR, where it is involved in redox sensing (26). Moreover, C114 has a position similar to C127 of *X. campestris* OhrR, which forms an intermolecular disulfide bond with the N-terminus cysteine in the oxidized regulator (26). As a first approach to studying the redox-sensing mechanism of SydR, we generated a structural model of the reduced SydR dimer, based on crystal structures of MarR-type regulators that are available in the Alphafold Protein Structure Database (Fig. 3B). The predicted distance between C16 and C114′ (13.2 Å) is similar to that determined between C22 and C127′ (15.5 Å) in the crystal structure of *X. campestris* OhrR (25). Based on this model, the two cysteines of SydR may be involved in the formation of an intermolecular disulfide bond and oxidative inhibition of SydR.

To investigate the role of C16 and C114 in the redox-regulated activity of SydR, cysteines were replaced with serine by site-directed mutagenesis, and recombinant proteins containing one or two mutated cysteines were produced. The covalent dimerization state of SydR was assessed by non-reducing SDS-PAGE using WT and mutant proteins, first reduced with DTT and then challenged or not with NaOCl (Fig. 3C). The reduced proteins migrate mainly as monomers with a molecular mass of 20.4 kDa. Upon NaOCl incubation, the monomers of SydR′ (CC) were totally converted into covalent dimers, indicating that the oxidant induces the formation of intermolecular disulfide bond(s). Moreover, the SydRC16SC114S (SS) derivative remained in the monomeric form while the SydRC114S (CS) and SydRC16S (SC) derivatives were greatly and weakly converted into dimers, respectively. These findings illustrate the formation of disulfide bonds between cysteines at corresponding positions in the monomers (i.e., between C16 and C16′, and C114 and C114′). However, the monomeric form predominates in oxidized SydRC16S (SC) and SydRC114S (CS) lanes, supporting an effective C16-C114′ disulfide bond in SydR. All these data suggest that both cysteines are involved in the covalent dimerization of SydR, with C16 playing a critical role.

The role of cysteine residues in DNA binding was also analyzed (Fig. 3D). EMSA assays showed that SydRC114S (CS) was still able to bind to the target DNA, whereas the C16 mutation in SydRC16S (SC) and SydRC16SC114S (SS) impaired DNA binding. Since C16 is located in helix α1, its substitution with Ser might impact SydR′ folding and consequently, non-covalent dimerization and DNA-binding properties of the protein.

To strengthen the DNA-binding analyses, a comparison of SMa2023 expression in *S. meliloti* strains overexpressing SydR or SydR derivatives was performed (Fig. 3E). The SMa2023 expression levels in Δ*sydR-*p*trp:sydRC114S* (CS) and Δ*sydR*-p*trp:sydR* strains were similar and dependent on NaOCl addition. In contrast, gene expression in Δ*sydR-*p*trp:sydRC16S* (SC) and Δ*sydR-*p*trp:sydRC16SC114S* (SS) was, respectively, 35-fold and 1,000-fold higher compared to untreated Δ*sydR*-p*trp:sydR*, regardless of the redox condition. These data confirm the importance of C16 in redox sensing, and the difference in SMa2023 induction between Δ*sydR-*p*trp:sydRC16S* (SC) and Δ*sydR-*p*trp:sydRC16SC114S* (SS) suggests a role for C114 in SydR activity.

Overall results demonstrate the importance of C16, together with a potential secondary role of C114 in SydR folding. They also show that SydR activity is reversibly inhibited by oxidation, and this redox regulation requires the conserved C16. Moreover, SydR displays DNA-binding activity that is more particularly modulated by NaOCl than H_2_O_2_ or tBOOH.

### Analysis of SydR involvement in ROS scavenging in free-living bacteria

**Fig 4:**
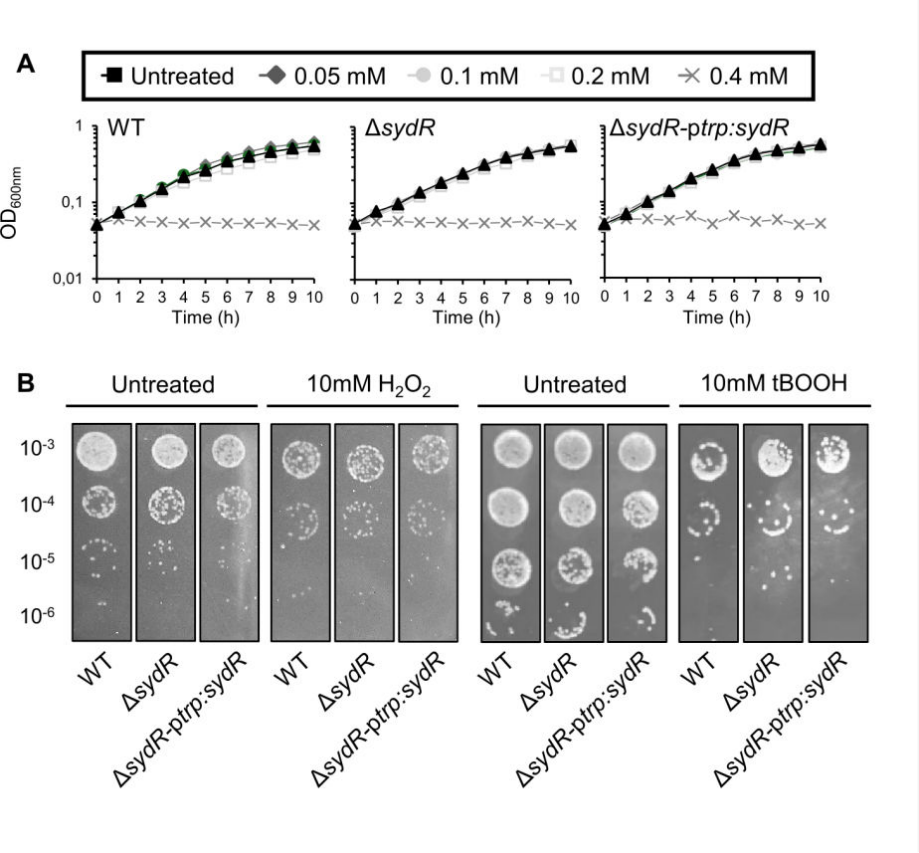
SydR inactivation has no effect on growth or oxidant sensitivity. (**A**) Growth of WT, Δ*sydR*, and Δ*sydR*-p*trp:sydR* strains in M9 + casamino acids (M9-CSA) in the presence of various concentrations of NaOCl. (**B**) Bacterial survival after challenge with H_2_O_2_ or tBOOH. WT and Δ*sydR* strains were grown in M9-CSA to OD_600_ of 0.1, then incubated in the presence of either H_2_O_2_ or tBOOH for 1 h before being serially diluted and spotted on agar plates. Data are representatives of at least three independent experiments.

As a redox-sensing regulator, SydR could be involved in the modulation of ROS detoxifying pathways. Thus, the effect of SydR inactivation on the sensitivity of *S. meliloti* to NaOCl, H_2_O_2_, or tBOOH was analyzed (Fig. 4). Exogenous concentrations of NaOCl up to 0.2 mM had no effect on the growth of the WT strain, while the addition of 0.4 mM NaOCl immediately stopped it. The behavior of Δ*sydR* and Δ*sydR*-p*trp:sydR* strains was not significantly different from that of the WT (Fig. 4A). Moreover, the ability of the three strains to survive after 1 h exposure to 10 mM H_2_O_2_ or tBOOH was similar (Fig. 4B). Thus, SydR was not involved in *S. meliloti* resistance to oxidative stress under our conditions.

The effect of *sydR* inactivation on bacteria challenged with ROS was further analyzed by using the genetically encoded biosensor roGFP2-Orp1. This redox probe proved to be an accurate tool for measuring dynamic changes in intracellular H_2_O_2_ pool in *S. meliloti* (8). The roGFP2-Orp1 is directly oxidized by H_2_O_2_, and it can also react with other peroxides and NaOCl, potentially responding to these oxidants in bacterial cultures (27, 28).

The basal oxidation level of roGFP2-Orp1 was similar in WT and Δ*syd*R strains, corresponding to a highly reducing redox potential EroGFP2 (−290 mV) as previously reported (8). Upon addition of oxidants, similar kinetics of roGFP2-Orp1 oxidation were observed in WT and Δ*sydR* mutant strains. Treatment with H_2_O_2_ (0.1–10 mM) resulted in dose-dependent oxidation of roGFP2-Orp1, followed by biosensor partial recovery within 10 min exposure (Fig. S2A). In cells treated with NaOCl (0.05–1 mM), the biosensor also experienced rapid and reversible oxidation (Fig. S2B). In contrast, roGFP2-Orp1 became highly oxidized within 10 min of adding tBOOH (0.05–5 mM) and could recover its reduced state when the treatment time was extended to 1 h (Fig. S2C and S3). These results showed the capacity of roGFP2-Orp1 expressed in *S. meliloti* to detect real-time changes in intracellular H_2_O_2_, as described earlier (8), together with tBOOH or NaOCl levels as previously shown for other bacteria (27, 28). However, the dynamics of these pools and the toxicity of the molecules were similar in WT and Δ*sydR* strains, revealing no major role of SydR in the control of ROS level in free-living bacteria.

### SydR plays a crucial role in the development of *S. meliloti*/*M. truncatula* symbiosis

**Fig 5:**
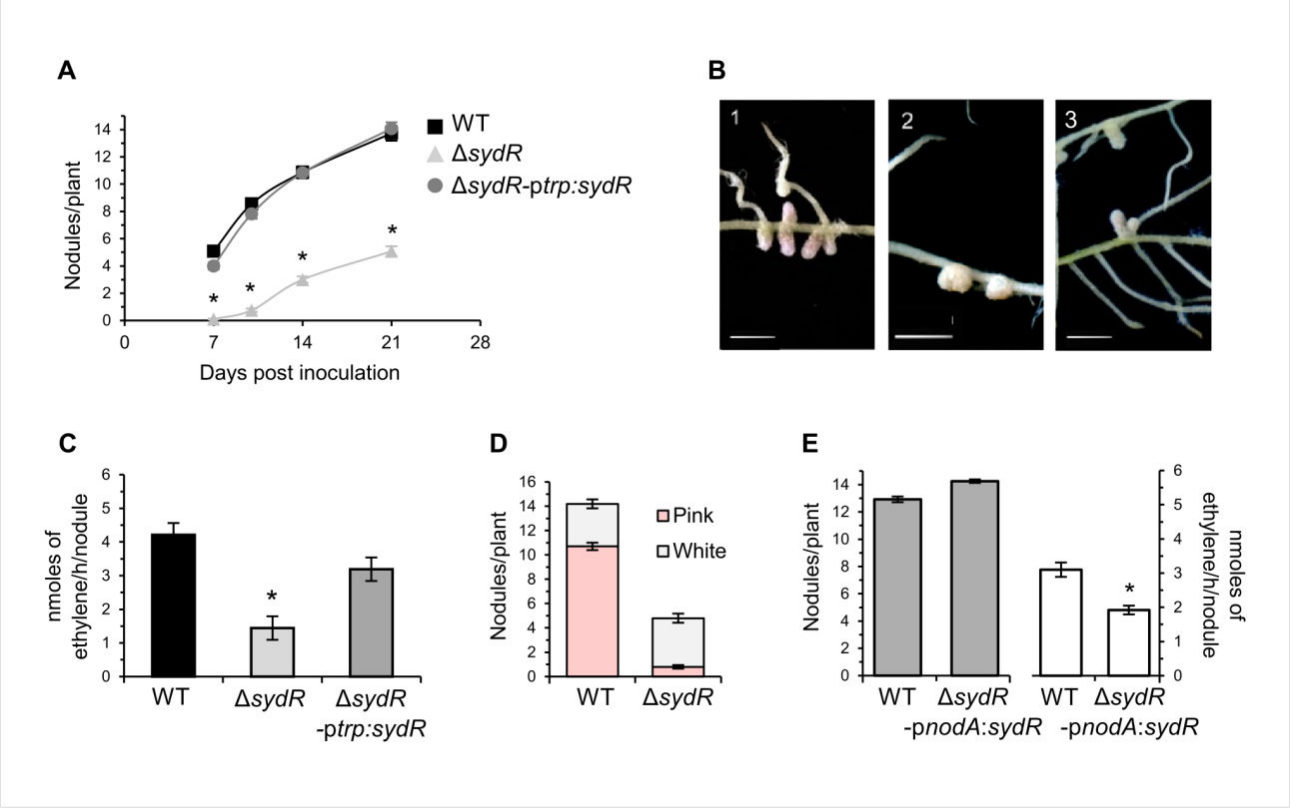
SydR plays a crucial role in *S. meliloti/M. truncatula* symbiosis. (**A**) Nodulation kinetics of *M. truncatula* plants inoculated with WT, Δ*sydR*, and Δ*sydR-*p*trp:sydR* strains. (**B**) Representative images of *M. truncatula* root nodules after inoculation with WT (1), Δ*sydR* (2), and Δ*sydR-*p*trp:sydR* (3). (**C**) Nitrogen fixation activity, determined by ARA at 21 dpi. (**D**) Number of white and pink nodules at 21 dpi. (**E**) Nodule number (left graph) and ARA (right graph) from WT (black bars) and Δ*sydR*-p*nodA:sydR* (white bars) inoculated roots at 21 dpi. The values shown are the means ± SEM of three independent experiments. Non-parametric Kruskal-Wallis and *post hoc* Conover-Iman tests with Benjamini-Hochberg correction (**A and C**), and Mann-Whitney test (**E**) were used to assess the statistical significance of differences compared to the WT-inoculated roots (*P < 0.05).

The symbiotic phenotype of Δ*sydR* mutant was analyzed during interaction with *M. truncatula* (Fig. 5) and *M. sativa* (Fig. S5). Nodulation tests and N_2_ fixation measurements [acetylene reduction assay (ARA)] were performed using plants inoculated with WT, Δ*sydR*, or Δ*sydR-*p*trp:sydR* strains. Inoculation of *M. truncatula* roots with Δ*sydR* mutant led to a drastic reduction of nodule number compared to the WT strain (Nod^−^ phenotype; Fig. 5A). Moreover, most *M. truncatula* nodules elicited at 21 days post-inoculation (dpi) by the mutant remained white and spherical as non-fixing nodules, while the majority of nodules infected by the WT and Δ*sydR-*p*trp:sydR* strains were pink and elongated (Fig. 5B). Consistently, nitrogen fixation in the nodules of roots inoculated with the Δ*sydR* mutant strain was reduced by 75% compared to those infected by the WT strain (Fix^−^ phenotype; Fig. 5C), and the remaining fixation most probably comes from the few pink nodules that have managed to develop (Fig. 5D). The constitutive expression of *sydR* in the Δ*sydR-*p*trp:sydR* strain restored the Nod^+^/Fix^+^ phenotype (Fig. 5C). These data showed that the *sydR* mutation drastically affects nodule development in the symbiotic interaction with *M. truncatula*.

To focus on the involvement of SydR in nodule functioning, we constructed a bacterial strain affected in *sydR* expression once released from ITs. This strain (Δ*sydR-*p*nodA:sydR*) carries *sydR* under the control of the p*nodA* promoter that is active from the beginning of interaction to bacterial release inside the plant cell (20). This strain formed elongated pink nodules similar to WT-induced nodules (Fig. 5E), where the expression of *sydR* and SMa2023 was downregulated and upregulated, respectively, compared with WT-induced nodules, as expected (Fig. S4). Moreover, at 21 dpi, the nodules infected by the Δ*sydR-*p*nodA:sydR* strain displayed a Fix^+/−^ phenotype with a 40% deficiency in the ARA (Fig. 5E). These results demonstrate that SydR activity contributes to both nodule formation and functioning. In symbiosis with *M. sativa*, roots inoculated by Δ*sydR* also formed less nodules than roots inoculated with WT, but the deficit is less drastic compared to *M. truncatula* roots (reduction of nodule number by 35% versus 63%, as compared to WT, at 21 dpi; Fig. S5A). Moreover, the nitrogen fixation capabilities of WT and Δ*sydR* infected nodules were similar (Fig. S5B).

### An essential role of SydR in the infection process

**Fig 6:**
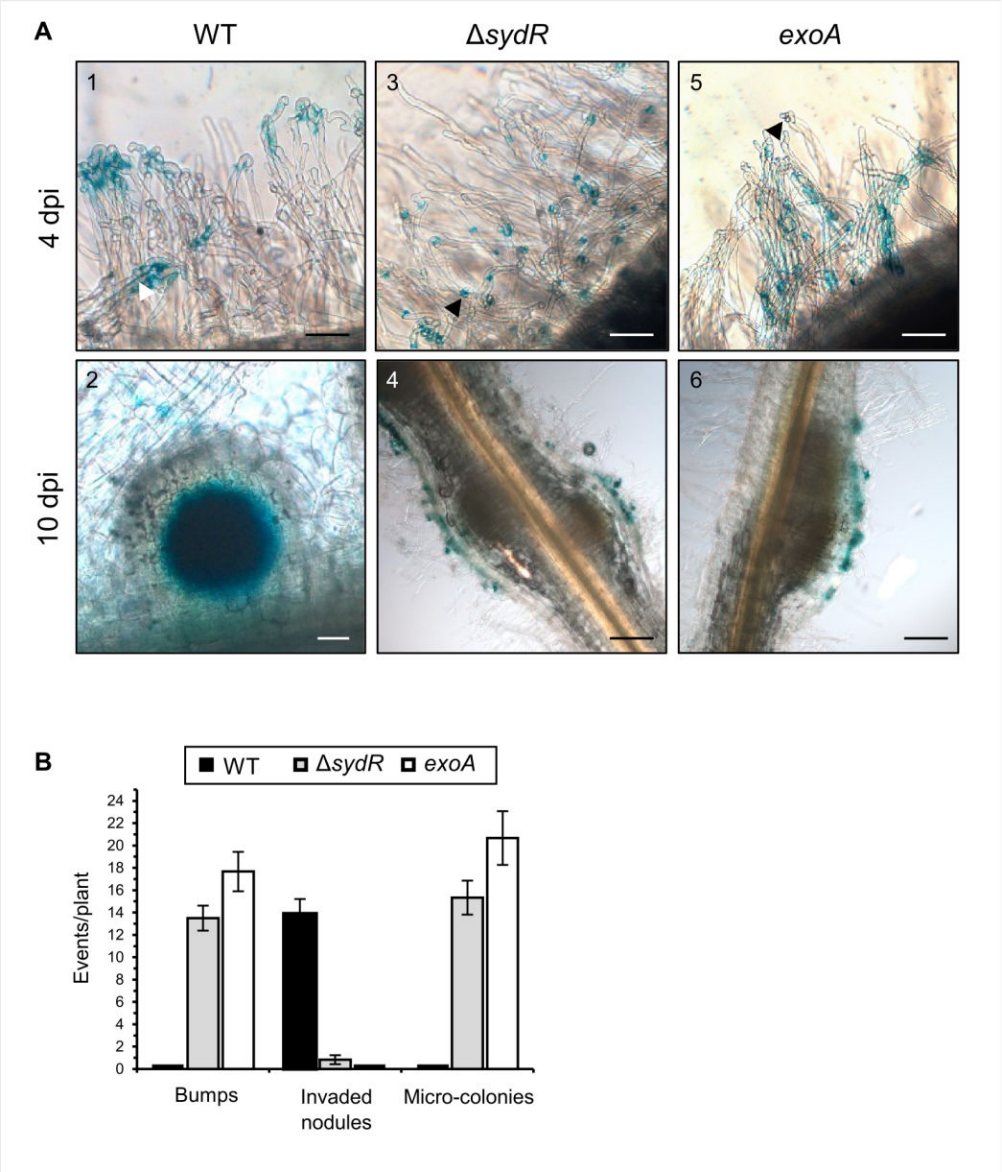
Δ*sydR* mutant is defective in root infection. (**A**) Light microscopic images of *M. truncatula* roots inoculated with the WT (1, 2), Δ*sydR* (3, 4), and *exoA* (5, 6) strains expressing p*hemA:lacZ* reporter fusion. β-Galactosidase activity (blue staining) was detected in (i) WT bacteria entrapped in root hairs (1), inside infection thread (white arrowhead) and nodule cells (2), and (ii) Δ*sydR and exoA* bacteria as micro-colonies mainly accumulated in RHCs (black arrowhead; 3, 5) and at the surface of bumps (4, 6). Scales: 50 µm (1, 2, 3, 5); 200 µm (4, 6). (**B**) Number of bumps, invaded nodules, and micro-colonies at 10 dpi. The values shown are the means ± SEM of three independent experiments. Significance compared to WT-inoculated roots was determined in Kruskal-Wallis and *post hoc* Conover-Iman tests with Benjamini-Hochberg correction (*P* < 0.05).

The step of root invasion affected by the *sydR* mutation in *M. truncatula* symbiosis was thereafter specified. Bacterial progression in roots was examined by using strains expressing *lacZ* under the control of a promoter highly expressed *in planta* (p*hemA:lacZ* transcriptional fusion). Roots were inoculated with WT, Δ*sydR* and, for comparison, with an *exoA* mutant known to form early aborted IT (29). In roots inoculated with WT, bacteria were detected within IT at 4 dpi, and readily visible inside nodules at 10 dpi (Fig. 6A, 1 and 2). In the case of roots inoculated with the Δ*sydR* mutant, *lacZ* staining at 4 dpi showed that bacteria mostly accumulate as micro-colonies inside root hair curling (RHC) (Fig. 6A, 3). These micro-colonies persisted at 10 dpi, on the surface of small nodule-like bumps devoid of bacteria (Fig. 6A, 4). In rare cases, infected nodules were also observed (Fig. 6B). The *exoA* mutant elicited only empty bumps with persistent micro-colonies on the surface (Fig. 6A, 5 and 6). These observations showed that the Δ*sydR* mutant is blocked at the initiation of infection, similar to the *exoA* mutant, once the infection pocket is formed.

**Fig 7:**
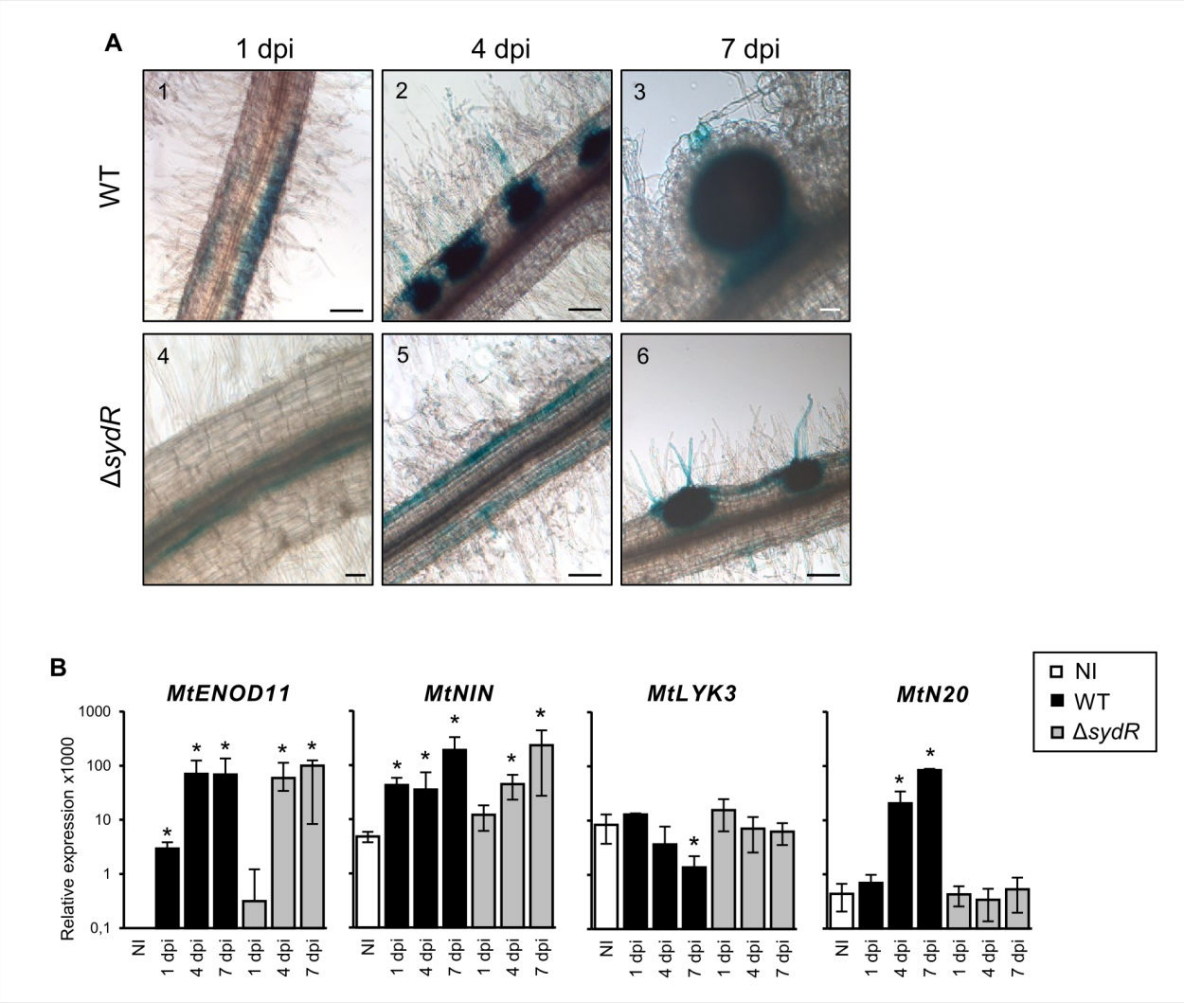
Root inoculation with Δ*sydR* mutant leads to delayed pre-infection and abortive infection events. (**A**) Light microscopic images of *M. truncatula* transgenic roots expressing p*MtENOD11-gusA* fusion inoculated with the WT and Δ*sydR* strains. GUS activity (blue staining) was visible in epidermal cells in the region of developing root hairs (1, 5) in activated cortical cells corresponding to the initial infection site (2, 6) and in invaded tissues of WT-induced nodules (3). Scales: 50 µm (3, 4); 200 µm (1, 2, 5, 6). (**B**) RT-qPCR analysis of the expression of *M. truncatula* genes in roots inoculated with either WT or Δ*sydR* at 1, 4, and 7 dpi. The expression ratios for marker genes relative to reference genes are shown. Expression level of genes in non-inoculated roots (NI) was also determined. The values shown are the means ± SEM of three independent experiments. Non-parametric Kruskal-Wallis and *post hoc* Conover-Iman tests with Benjamini-Hochberg correction were used to assess the statistical significance of differences between inoculated and non-inoculated roots (*P < 0.05).

The response to NFs and initiation of root infection were analyzed using plants expressing the reporter fusion p*MtENOD11-gusA*, and in RT-qPCR experiments. *MtENOD11* is an early nodulin gene expressed firstly in the root epidermis, later in the cortex around the infection threads, and then in the central tissue of young nodules (30). As previously described**,** blue staining corresponding to glucuronidase (GUS) activity was detected in transgenic roots inoculated with the WT strain: (i) in epidermal cells within 1 dpi, (ii) restricted to infected root hairs and activated cortical cells within 4 dpi, and (iii) later in the core tissue of young nodules (Fig. 7A, 1–3). By comparison, the expression of *GUS* fusion in roots inoculated with the Δ*sydR* mutant was detected in epidermal cells at 4 dpi, and then in cortical infection zones at 7 dpi (Fig. 7A, 4–6). These observations show a delayed induction of *MtENOD11* expression in roots inoculated with the mutant strain, indicating an alteration of the plant response. In parallel, changes in NF-dependent gene expression associated with pre-infection step (*MtNIN*, a key regulator of the NF pathway, in addition to *MtENOD11*), or linked directly to the infection process (*MtLYK3* and *MtN20*) were investigated by RT-qPCR (31–33). As shown in Fig. 7B, *MtNIN* and *MtENOD11* increased upon inoculation with WT and Δ*sydR* mutant strains. However, the induction of both genes occurs significantly later in Δ*sydR* mutant as compared to WT (4 dpi versus 1 dpi). In addition, the expression level of *MtLYK3* decreased, while *MtN20* increased, in roots inoculated with WT strain but remained constant over 7 dpi in those inoculated with the mutant strain.

Altogether, these data show that the NF-induced signaling pathway is active during the interaction between *M. truncatula* and the Δ*sydR* mutant strain. However, the transcriptional response of the plant and root colonization are altered by the mutation.

## DISCUSSION

In this work, we characterized a new thiol-based redox sensor of *S. meliloti*, SydR, which is critical for the establishment of symbiosis with *M. truncatula*. SydR is one of the 17 proteins annotated as MarR family transcriptional regulators in the *S. meliloti* Genome Database. A high number of MarR-type regulators, among others, is a typical feature of bacteria that adopt different lifestyles and have to respond to environmental changes (34, 35). Indeed, several MarR family members have been shown to play a major role in various symbiotic or pathogenic interactions with plants, such as *S. meliloti* WggR and *Dickeya dadantii* PecS (36–39). To a lesser degree, OhrR of *Azorhizobium caulinodans* has also been involved in a symbiotic interaction with host plants (40). SydR is the first redox-sensing MarR-type regulator shown to play a crucial role in the interaction with *M. truncatula*.

Like most MarR-type regulators, SydR represses transcription of the adjacent gene (SMa2023). This repression (~1,000-fold) is variously relieved by the addition of NaOCl, H_2_O_2_ or tBOOH. Indeed, in the WT strain, the transcription of SMa2023 was increased ~100-fold and approximately twofold upon treatment with NaOCl and H_2_O_2_/tBOOH, respectively, showing that NaOCl is a more potent inducer than hydroperoxides for the expression of SydR target gene. In comparison, the expression of OhrR target genes, *ohrA* in *Bacillus subtilis,* and *ohr* in *S. meliloti*, is similarly induced by NaOCl and organic hydroperoxides (this study, Fig. S1) (41, 42).

Our data furthermore demonstrated that NaOCl has a direct effect on SydR *in vitro* and leads to its release from the operator DNA. They point out that, between the two cysteines of SydR, the conserved C16 alone is essential for redox sensing and engaged in an intermolecular disulfide bond. The two cysteines are located on the α1 and α5 helices, which contribute to the formation of a dimer interface. As shown for many MarR-type regulators, oxidation of the N-terminal sensor cysteine may induce allosteric conformational changes of the DNA-binding domain and lead to dissociation from DNA (10).

Up to now, NaOCl was shown to trigger either the formation of an intermolecular disulfide bond between two distinct cysteines, i.e., in *B. subtilis* YodB and HypR, or S-thiolation of the single redox-sensitive cysteine of *B. subtilis* OhrR (42–44). Besides, oxidative inactivation of a few MarR-type regulators has been shown to involve an intersubunit disulfide bond between the single cysteine residues of both subunits (the 1-Cys type QorR of *Corynebacterium glutamicum*, HypS of *Mycobacterium smegmatis*, and PecS of *Pectobacterium atrosepticum* [45–47]). Our *in vitro* and *in vivo* analyses suggest that the oxidation of SydR protein involves different mechanisms, depending on the integrity of the C114. Non-reducing SDS-PAGE analysis revealed a covalent dimerization for the SydRC114S (CS) derivative, necessary involving C16-C16′ disulfide bond. Nevertheless, this dimerization is partial in SydRC114S (CS), while the dimerization is complete in SydR (CC). This strongly suggests that the dimerization of SydR occurs mainly by the formation of C16-C114′ disulfide, which is consistent with the structural model of SydR (Fig. 3B).

Further analyses are required to unravel the structural mechanisms for the inactivation of SydR upon oxidation. In particular, resolving the structure of reduced and oxidized SydR would help elucidating the thiol-oxidation mechanism of C16 and associated conformational changes.

These data highlight the role of SydR as a redox switch under hypochlorite/NaOCl and, potentially, hydroperoxide-induced oxidative stress. The physiological signal regulating SydR activity during interaction remains to be determined. Hypochlorite, which is produced by the animal immune system (48), has not been detected in plants. On the other side, H_2_O_2_ is present during nodule development and functioning (9), while organic peroxide is most probably present in mature nodules (9, 16). Both oxidants might play a role in SydR regulation *in planta*.

Thiol-disulfide switches in redox-sensing regulators mainly result in the activation of specific detoxification pathways to restore cellular redox homeostasis upon stress conditions (1). Our data on SydR, both toxicity assays and kinetic analyses of roGFP2-Orp1 oxidation, suggested that the regulator does not play a significant role in response to exogenous H_2_O_2_, tBOOH, or NaOCl. Maybe a difference in ROS sensitivity could be observed by inactivating SydR target(s) rather than *sydR* itself. This has been exemplified by the work of Fontenelle et al. (16), where the inactivation of *ohr*, but not *ohrR*, modified the sensitivity of *S. meliloti* to oxidant.

SydR is required for the infection of *M. truncatula*. Firstly, the Δ*sydR* mutant gives a drastically reduced number of nodules compared to WT, and generates non-invaded bumps instead. Secondly, microscopic and RT-qPCR analyses showed that early NF signaling, cortical cell activation, and nodule primordium initiation still arise in roots inoculated with Δ*sydR*. Similar observations were obtained with the *exoA* mutant blocked at the stage of IT initiation [our data (49)]. Likewise, the repression of *MtLYK3* expression, which is directly linked to infection, did not occur in roots inoculated by one or the other mutant (31, 33). Finally, it can be assumed that the infection process in Δ*sydR-*inoculated roots arrests earlier than the process in *exoA-*inoculated roots since, unlike the latter, it lost the ability to trigger *MtN20* induction. Inoculation with the Δ*sydR* mutant, moreover, led to a delayed induction of *MtENOD11* and *MtNIN* expression. Due to lack of infection, most of the cortical cells activated by Δ*sydR* inoculation may no longer be susceptible to signals that induce further nodule development. Finally, by complementing only the infection step, it was possible to show the involvement of SydR in the optimal nodule functioning. For example, SMa2023 product, a putative periplasmic lipase, is expressed in differentiated bacteroids.

By contrast, Δ*sydR* mutant induces the formation of nitrogen-fixing nodules with *M. sativa*. These data are similar to the results of Zhang et al. (17) with a SMa2020 deletion mutant generated in Rm1021 background. They show the importance of plant species (*M. truncatula* versus *M. sativa*) in the outcome of the interaction. Indeed, distinct phenotypic effects across different genetic backgrounds have been already observed such as the formation of non-invaded bumps and effective nodules on, respectively, *M. truncatula* and *M. sativa* roots, inoculated with the same *S. meliloti* lipopolysaccharide mutant (23, 50). As another example, inactivation of the *relA* gene involved in the stringent response was shown to affect the capacity of *S. meliloti* to infect *M. sativa* only (51). Thus, it can be assumed that checkpoints involved in bacterial root infection function differentially in *M. truncatula* and *M. sativa*. Most likely, genotypic and phenotypic analyses of bacteria isolated from the rare pink nodules produced by Δ*sydR* in *M. truncatula* roots will help identify functions specifically involved in *S. meliloti/M. truncatula* interaction.

Further studies will be required to identify the mechanisms/targets controlled by SydR, particularly those whose fine regulation is required for the symbiosis establishment and functioning.

## MATERIALS AND METHODS

### Bacterial strains and growth conditions

All bacterial strains and plasmids used in this study are listed in Table S2. *Escherichia coli* strains were grown at 37°C in Luria-Bertani (LB) medium. *S. meliloti* strains were grown at 30°C in LB medium supplemented with 2.5 mM MgSO_4_ and 2.5 mM CaCl_2_ (LBMC), in M9 medium and in M9-CSA with appropriate antibiotics, as specified in Text S1 in the Supplemental Material.

### Preparation of bacterial samples for RT-qPCR analysis and toxicity tests

Cultures of *S. meliloti* strains were grown in M9 to mid-log phase (OD_600_ of ~0.3), then divided into 10 mL aliquots and exposed to various oxidants for 10 min (1 mM H_2_O_2_; 200 µM tBOOH; 50 µM plumbagin; 0.2 mM NaOCl) or 30 min (25 µM spermine NONOate). The bacterial cultures were immediately centrifuged, and the pellets were frozen at *−*80°C until RNA extraction. For testing oxidant toxicity, cultures grown to mid-log phase in M9-CSA were diluted to an OD_600_ of ~0.1, then divided into 5 mL aliquots that were incubated with 1 mM H_2_O_2_ or 10 mM tBOOH for 1 h, or further grown with various concentrations of NaOCl. Samples treated with H_2_O_2_ or tBOOH were serially diluted, and 10 µL was spotted in three replicates on LBMC plates; colony-forming units (CFU) were counted after 48 h of incubation at 30°C. The effect of NaOCl was evaluated by monitoring OD_600_ for 6 h. An untreated culture was included as a control in each experiment. Three independent biological repetitions were performed for each assay.

### Plant growth conditions

*M. truncatula* ecotype Jemalong A17 and *M. sativa* L. var. Europe (alfalfa) were used as the host plants to test nodulation and nitrogen fixation of *S. meliloti* strains. Seed germination and plant growth procedures were performed as described previously (52, 53) with modifications summarized in Text S1.

### Construction of *S. meliloti* mutants

The ΔSMc03824, SMc00146, and Δ*sydR* mutants were constructed using allelic exchange mutagenesis, as described in Text S1.

### Molecular cloning and mutagenesis of *sydR*

Primers used for DNA amplification are listed in Table S3. Molecular cloning and mutagenesis of *sydR* were performed as described in Text S1.

### Real-time measurements of intracellular redox potential

Variations in intracellular redox potential of *S. meliloti* were monitored with ratiometric roGFP2 fluorescence measurements (excitation at 405 and 488 nm wavelengths, emission at 515 nm) using a spectrofluorometer/luminometer (Xenius, Safas, Monaco), as previously described (8).

### RNA extraction and RT*-*qPCR assays

RNA extraction and analysis were performed as described in Text S1.

### Purification of SydR wild type and mutant proteins

The production and purification of recombinant proteins were performed as described in Text S1.

### Intersubunit disulfide bond assay

SydR′ and mutant proteins were reduced in 100 mM DTT for 2 h and desalted using ultra centrifugal columns with 10 NMWL. The proteins were oxidized by treatment with 100 mM NaOCl for 30 min. Additionally, 100 mM N-ethylmaleimide was applied to each sample for 2 h at room temperature to reduce the formation of non-specific disulfide bonds. Finally, the samples were analyzed by 10% non-reducing SDS-PAGE.

### EMSA

For EMSA analysis, the 144 bp DNA fragment covering the *sydR*-SMa2023 intergenic region was amplified using *sma2020*_23F/*sma2020*_23R primers, and a 138-bp internal fragment of SMa2019 used as non-target DNA was amplified using *sma2019* up114/*sma2019* down251 primers. DNA-binding reactions were implemented in a binding buffer (10 mM Tris-HCl [pH 8], 50 mM KCl, 0.5 mM EDTA, 10% glycerol, 0.5 mM DTT) containing various amounts of SydR′ WT protein or mutant variants and 10 nM DNA. EMSA was conducted with SydR′, either subjected to different oxidant treatments or left untreated, at 25°C for 25 min. Subsequently, an extra 25-min incubation with 2 or 4 mM DTT was performed for samples as required. The samples were loaded on 8% polyacrylamide gel in 0.5× Tris borate-EDTA buffer. The Diamond Nucleic Acid Dye (Promega) staining was used to visualize DNA on the gel. The gel was analyzed under UV light (UVIDOC HD6, Cambridge).

### Nitrogen fixation assays

N_2_-fixation activity was determined at 21 dpi by assessing C_2_H_2_ reduction using gas chromatography (Agilent Technologies 6890N) as previously described (53). At least 50 plants from three independent biological repetitions were analyzed for each inoculation.

### Microscopy and histology analysis

Roots expressing the p*MtENOD11-gusA* fusion were harvested at 1, 4, and 7 dpi (*n* = 4 roots per time point). Roots inoculated with bacterial strains expressing the p*hemA:lacZ* fusion were harvested at 4 and 10 dpi (*n* = 6 roots per time point), or 14 dpi. GUS and β-galactosidase staining assays were performed as described previously (30, 54) with modifications summarized in Text S1. Stained roots and nodule sections were observed under a transmission light microscope (Zeiss Axioplan II).

## SUPPLEMENTAL MATERIAL

Supplemental Material mbio.02275-24-s0001.pdf Additional experimental details and supplemental tables and figures.

ASM does not own the copyrights to Supplemental Material that may be linked to, or accessed through, an article. The authors have granted ASM a non-exclusive, world-wide license to publish the Supplemental Material files. Please contact the corresponding author directly for reuse.
